# Clinical trial data sharing: a cross-sectional study of outcomes associated with two U.S. National Institutes of Health models

**DOI:** 10.1038/s41597-023-02436-0

**Published:** 2023-08-08

**Authors:** Anisa Rowhani-Farid, Mikas Grewal, Steven Solar, Allen O. Eghrari, Audrey D. Zhang, Cary P. Gross, Harlan M. Krumholz, Joseph S. Ross

**Affiliations:** 1 Department of Practice, Sciences, and Health Outcomes Research, University of Maryland, Baltimore, 220 N Arch St., Baltimore, MD 21201 USA; 2 Section of General Internal Medicine, Yale School of Medicine, 333 Cedar St., New Haven, CT 06510 USA; 3 Genome Informatics Section, Computational and Statistical Genomics Branch, National Human Genome Research Institute, National Institutes of Health, 31 Center Dr, Bethesda, MD 20894 USA; 4 Johns Hopkins University School of Medicine, 600 N Wolfe St, Baltimore, MD 21287 USA; 5 Department of Internal Medicine, Duke University School of Medicine, 2301 Erwin Rd., Durham, NC 27710 USA; 6 Cancer Outcomes Public Policy and Effectiveness Research (COPPER) Center, Yale School of Medicine, 367 Cedar St., New Haven, CT 06520 USA; 7 National Clinician Scholars Program, Yale School of Medicine, 333 Cedar St., New Haven, CT 06510 USA; 8 Center for Outcomes Research and Evaluation (CORE), Yale-New Haven Hospital, 1 Church St., Suite 200, New Haven, CT 06510 USA; 9 Section of Cardiovascular Medicine, Yale School of Medicine, 333 Cedar St., New Haven, CT 06510 USA; 10 Department of Health Policy and Management, Yale School of Public Health, 60 College St., New Haven, CT 06520 USA

**Keywords:** Research data, Outcomes research

## Abstract

The impact and effectiveness of clinical trial data sharing initiatives may differ depending on the data sharing model used. We characterized outcomes associated with models previously used by the U.S. National Institutes of Health (NIH): National Heart, Lung, and Blood Institute’s (NHLBI) centralized model and National Cancer Institute’s (NCI) decentralized model. We identified trials completed in 2010–2013 that met NIH data sharing criteria and matched studies based on cost and/or size, determining whether trial data were shared, and for those that were, the frequency of secondary internal publications (authored by at least one author from the original research team) and shared data publications (authored by a team external to the original research team). We matched 77 NHLBI-funded trials to 77 NCI-funded trials; among these, 20 NHLBI-sponsored trials (26%) and 4 NCI-sponsored trials (5%) shared data (OR 6.4, 95% CI: 2.1, 19.8). From the 4 NCI-sponsored trials sharing data, we identified 65 secondary internal and 2 shared data publications. From the 20 NHLBI-sponsored trials sharing data, we identified 188 secondary internal and 53 shared data publications. The NHLBI’s centralized data sharing model was associated with more trials sharing data and more shared data publications when compared with the NCI’s decentralized model.

## Introduction

Clinical trial data sharing involves sharing data generated from a clinical trial, which includes protocols, informed consent forms, case report forms, clinical study reports, and individual patient-level data (IPD). Sharing clinical trial data has numerous benefits, including creating opportunities to pursue additional research questions, meta-analyses, and independent verification and reproducibility of study results^1–3^. Data sharing also aims to maximize the value of clinical trial data by strengthening the quality and totality of the evidence driving medical decisions^4^. In recent years, sharing clinical trial data, particularly IPD, has become more commonplace among scientific researchers and sponsors, catalyzing the development of various models for sharing IPD^5–9^.

IPD data sharing may occur through one of several frameworks^8^. In a centralized model, IPD are prepared and released into a central repository with access overseen by an independent entity, commonly a private or public funder, foundation, or academic institution, that reviews and approves data requests; IPD is then sent to investigators^10^. Alternatively, in a decentralized model, investigators independently or as part of a research collaborative, such as a cooperative research group, retain control of their data and grant access to data requests at their discretion. A third approach involves unrestricted public availability, where IPD are made freely available to independent researchers without a gatekeeper in a repository; examples of these include Dryad, Figshare and Github. Due to patient privacy issues, it is not common for such publicly available data repositories to store IPD from clinical studies. As such, clinical trial data are usually shared via a decentralized or centralized model.

The NIH has been a long-standing proponent of data sharing, instituting an initial data sharing policy in 2003 requiring investigators to submit detailed data sharing plans as a condition for funding clinical trials, regardless of size, requesting $500,000 or more in direct costs in any year of the proposed project, and finalizing a data sharing policy in 2020 that became effective in 2023, requiring all NIH-funded research to submit a Data Management and Sharing Plan^11–14^. While NIH strongly encourages IPD sharing as part of its 2023 policy, it is not required. Instead, it is expected that when researchers design their required Data Management and Sharing Plans, they integrate data sharing into the routine conduct of research.

As investigators are increasingly considering the sharing of data generated from clinical trials, characterizing the outcomes of differing data sharing models and their impact will help inform future data sharing model structures and policies^15^. The NHLBI and NCI, as two examples of NIH Institutes sharing data, differ in their approaches to data sharing despite both being part of the NIH. For example, the NHLBI has had a data-sharing policy in place since 1989 that resulted in the creation of the NHLBI Data Repository in 2000, a centralized model for housing clinical data from NHBLI-sponsored studies^11^, managed under the NHLBI Biologic Specimen and Data Repository Information Coordinating Center (BioLINCC) since 2008^16^. Through BioLINCC, NHLBI-sponsored investigators with 500 or more participants in their studies or those requesting $500,000 or more in direct costs in any year of the proposed project must deposit IPD into a central repository; all data sharing requests are reviewed by NHLBI staff and are evaluated based on the inclusion of a description of the research plan/protocol, as well as documentation of review or or an exemption from review from an Institutional Review Board or Ethics Committee^11^.

In contrast, up until February 2017, NCI had adopted a more de-centralized approach to data sharing, relegating control to large cooperative groups and their alliances responsible for managing the data collected through their own studies. Each cooperative group maintained an electronic database housed at the Group’s Statistical Center that aggregated data from participating institutions^17^. The American College of Surgeons Oncology Group is an example of a cooperative group, while the Cancer and Leukemia Group B is another. In 2011, these two groups along with the North Central Cancer Treatment Group merged to formed the Alliance for Clinical Trials in Oncology. This Alliance has its own data and statistical center, the Alliance Statistics and Data Management Center and external investigators can request access to data through this Center. Before the formation of this Alliance, each Cooperative Group had their own statistical center and outside investigators could request IPD by submitting a formal request to the Group, which then could grant approval after internally reviewing the “scientific merits and feasibility” of the research proposal. Requests were considered only for data for which the primary study analyses had already been published^17^.

While the NCI data sharing model has since been updated to more closely approximate NHLBI’s through the creation of the NCTN/NCORP Data Archive which houses NCI-funded clinical trial data that were published on or after January 1, 2015 onwards^18^, the initial use of two different models by these NIH institutes allows for their comparison to determine their relative outcomes^19^.

The true value in sharing data lies in the generation and dissemination of new knowledge resulting from shared data. We defined these papers as secondary publications, those reporting IPD analyses in a peer-reviewed publication that were separate from that reported in the primary publication. We further categorized these secondary publications into two distinct groups: “shared data publications”, peer-reviewed research studies based on independent analyses of shared IPD and authored by investigators external to the primary study team, and “secondary internal publications”, peer-reviewed publications that were also based on independent analyses of shared IPD but where one or more authors from the original research team collaborated and were listed as authors.

Accordingly, we characterized the outcomes associated with the NHLBI and NCI models of data sharing, determining the rate of data sharing, and for those trials that shared data, the number of shared data publications and secondary internal publications and their metrics of research value, namely, their citation counts, h-index of the publishing journal, abstract views and Altmetric Attention Scores, for a matched sample of completed trials sponsored by each institute that were completed in 2010–2013. Evaluating the “outcomes” of existing initiatives via examination of secondary publications will aid in understanding how and to what extent IPD are being shared and used.

## Results

We identified 240 trials (84 NHLBI-funded and 156 NCI-funded) with a primary completion date between January 1, 2010 and December 31, 2013 that had enrolled 500 or more patients and/or had total costs greater than or equal to $1,000,000 in any given fiscal year. Of these, 29 (7 NHLBI-funded and 22 NCI-funded) were excluded as, according to ClinicalTrials.gov, 4 NHLBI-funded trials listed their status as unknown, 2 were terminated and 1 was still recruiting and 13 NCI-funded trials were terminated, 3 listed their status as unknown, 3 were sponsorded by external funders, 2 were withdrawn, and 1 was no longer available.

**Fig. 1:**
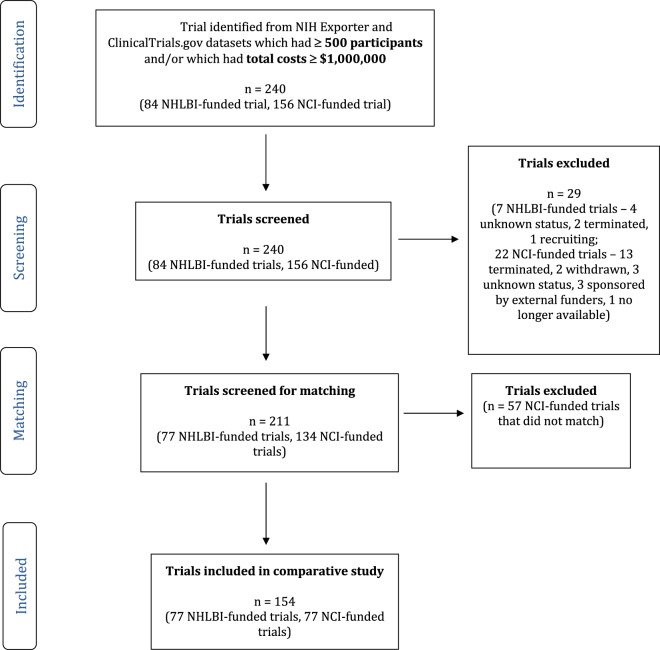
Flow diagram of selection of trials included in the comparative study.

Therefore, there were 77 NHLBI-sponsored trials and 134 NCI-sponsored trials with a primary completion date between January 1, 2010 and December 31, 2013 that listed their status as completed and eligible for data sharing, either because of total costs greater than or equal to $1,000,000 or enrollment of 500 or more patients. We matched the 77 NHLBI-funded trials to 77 NCI-funded trials that had similar completion dates, sample size and costs; see flow diagram depicted in Fig. 1. Among the 77 NHLBI-sponsored trials, 41 were interventional and 36 observational, whereas among the 77 NCI-sponsored trials, 53 were interventional and 24 observational; Table 1 summarizes the characteristics of the 154 trials, stratified by funder.

**Table 1:** Characteristics of the matched trials, stratified by funder.

Trial Characteristics	Funder				
	NHLBI (n = 77)		NCI (n = 77)		Significance
	*n*	%	*n*	%	
**Sample size**					
1–1000	45	58	44	57	p = 0.30^1^
1001–2000	15	19	23	30	
2001–3000	8	10	4	5	
>3001	9	12	6	8	
**Total Costs**					
<1,000,000	52	68	38	49	p = 0.07^2^
≥1,000,000	13	17	19	25	
Cost data not available	12	16	20	26	
**Study type**					
Observational	36	47	24	31	p = 0.07^3^
Interventional	41	53	53	69	

Characteristics of trials stratified by funder

^1^X2 = 3.63

^2^X2 = 5.30

^3^Fisher’s exact test value = 0.07.

### Results reporting, publication, and sharing of individual patient-level data

#### NHLBI-funded trials

Among the 77 NHLBI-funded trials, 16 (21%) reported their primary results on ClinicalTrials.gov, and 67 (87%) had their primary results published. The median time from primary study completion to result dissemination through a primary publication was 1.8 years (IQR, 1.2–2.9 years). In total, 20 of 77 (26%) NHLBI-funded trials shared their data. IPD were available for request on the BioLINCC website for 11 of 77 trials. 5 of 11 authors who were contacted clarified in their responses that data were not shared, 1 of 11 authors indicated that data was shared at the database for Genotypes and Phenotypes (dbGap) and we did not hear back from the remaining 5 of 11 authors (2 emails bounced back).

#### NCI-funded trials

Among the 77 NCI-funded trials, 18 (23%) reported their primary results on ClinicalTrials.gov, and 62 (81%) had their primary results published. The median time from primary study completion to result dissemination though a primary publication was 2.4 years (IQR, 1.1–3.7 years). A total of 4 of 77 (5.2%) trials shared data. One trial shared its data at the National Clinical Trials Network/NCI’s Community Oncology Research Program (NCTN/NCORP) Data Archive. Personal communication with 14 authors of NCI-funded studies confirmed that 2 trials had shared their data with other authors/institutions who were conducting meta-analyses requiring raw data. 5 of 14 authors clarified in their responses that data were not shared and we did not hear back from 7 of 14 authors (one email bounced back).

Overall, NHLBI-funded trials were more likely to share data when compared with NCI-funded trials (Odds Ratio = 6.4, 95% CI, 2.1–19.8; p = 0.001).

### Secondary internal publications and shared data publications

From the 20 NHLBI-funded trials sharing data, a total of 188 secondary internal publications with a median of 3 (IQR, 0–6) per trial were identified, along with 53 shared data publications with a median of 7 (IQR, 4–11) per trial. Among these 20 trials sharing data, 15 (75%) were found to have at least one secondary internal publication, and 6 (30%) were found to have at least one shared data publication.

From the 4 NCI-funded trials that shared data, a total of 65 secondary internal publications with a median of 7 (IQR, 2–21) per trial were identified, along 2 shared data publications with a median of 1 (IQR, 0–1) per trial. All 4 (100%) trials had secondary internal publications and 2 had associated shared data publications (one each, both of which were meta-analyses). These two trials were the *Disease Management for Smokers in Rural Primary Care* trial and the *Enhancing Tobacco Use Treatment for African American Light Smokers trial*. Table 2 reports these key outcomes, stratified by funder.

**Table 2:** Results reporting, data sharing, and publication practices of trials funded by NHLBI and NCI.

Key Outcomes	Funder	
	NHLBI (n = 77)	NCI (n = 77)
Trials reporting primary results on ClinicalTrials.gov (%)	16 (21%)	18 (23%)
Trials publishing primary results (%)	67 (87%)	62 (81%)
Median (IQR) time (years) from study completion to disseminating results through publication	1.8 (1.2–2.9)	2.4 (1.1–3.7)
Trials sharing data (%)	20 (26%)	4 (5%)
Of those trials sharing data, trials with shared data publications (%)	6 (30%)^1^	2 (50%)^2^
Of those trials sharing data, median (IQR) number of shared data publications	7 (4–11)	1 (1-1)
Of those trials sharing data, trials with secondary internal publications (%)	15 (75%)^1^	4 (100%)^2^
Of those trials sharing data, median (IQR) number of secondary internal publications	3 (0–6)	7 (2–21)

^1^The denominator here is the number of trials sharing data (20), not the total number of trials (77).

^2^The denominator here is the number of trials sharing data (4), not the total number of trials (77).

In our analyses, we intended to compare the impact of the two data sharing policies by examining shared data publications resulting from each funding model on the basis of metrics of research value, namely, their citation counts, h-index of the publishing journal, abstract views and Altmetric Attention Scores. However, the low rate of shared data publications among NCI-funded trials (resulting from only 2.6% of NCI-funded trials in the study sample) provided inadequate statistical power to discern differences between the two groups for these measures. For instance, the data demonstrated a non-significant trend favoring NHLBI-funded trials, with approximately 3.2 (95% CI: 0.62–16.2, p = 0.17) greater odds of the generation of shared data publications compared to NCI-funded trials. Neverthelss, to better illustrate the the potential research value of shared data publications, we further evaluated the NHLBI-funded trial that had produced the most number of shared data publications: the TOPCAT trial. For this trial and all the other trials that had shared data publications, we have publicly shared the dateset (10.17605/OSF.IO/T8B6G)^20^ which includes the characteristics of the secondary internal and shared data publications, including study types and impact measures.

#### Case Study of the Aldosterone Antagonist Therapy for Adults With Heart Failure and Preserved Systolic Function (TOPCAT) trial

The TOPCAT trial evaluated the effects of spironolactone in patients with heart failure and preserved systolic function. The trial found that spironolactone failed to reduce the incicdence of the composite primary outcome of death from cardiovascular causes, aborted cardiac arrest, or hospitalization for heart failure. The trial’s primary findings were published in *The New England Journal of Medicine* in April 2014 and the trial’s primary investigators shared its data at BioLINCC in April 2016.

The TOPCAT trial had a total of 37 secondary publications, 14 secondary internal publications and 23 shared data publications. Of the 14 secondary internal publications, 11 reanalyzed the primary outcome, 2 were secondary outcome studies and 1 was a pooled analysis. These secondary internal publications had median citation count of 22 (IQR 18.3–41.3), median h-index (of the publishing journal) of 123 (IQR 52.3–156), median abstract views of 4 (IQR 0–10.8) and median Altmetric Attention Score of 7 (IQR 2.8–18.8). Among the 23 shared data publications, 15 reanalyzed the primary outcome, 5 were secondary outcome studies, 2 were secondary analyses and 1 was a pooled analysis. These shared data publications had median citation count of 13 (IQR 4–26), median h-index (of the publishing journal) of 110 (IQR 55–118), median abstract views of 0 (IQR 0–11) and median Altmetric Attention Score of 4 (1–12). Table 3 summarizes these findings of the trial’s impact.

**Table 3:** Types of analyses and impact measures of additional internal publications and shared data publications for the NHLBI-funded trial: Aldosterone Antagonist Therapy for Adults With Heart Failure and Preserved Systolic Function (TOPCAT).

Types of analyses and impact measures	Publication Type	
	Additional internal publications (n = 14 publications)	Shared data publications (n = 23 publications)
Analysis type	11 reanalysis of primary outcome, 2 secondary outcome studies, 1 pooled analysis	15 reanalysis of primary outcome, 5 secondary outcome studies, 2 secondary analyses, 1 pooled analysis
Median (IQR) citation count	22 (18.3–41.3)	13 (4–26)
Median (IQR) h-index of publishing journal	123 (52.3–156)	110 (55–118)
Median (IQR) abstract views	4 (0–10.8)	0 (0–11)
Median (IQR) Altmetric Attention Score	7 (2.8–18.8)	4 (1–12)

## Discussion

In this matched sample of NIH-funded clinical trials completed in 2010–2013 with total costs greater than or equal to $1,000,000 in any given fiscal year or total enrollment of 500 or more patients, we found that NHLBI-funded trials had significantly higher odds of sharing data compared to NCI-funded trials. A trend toward an increased rate of shared data publications associated with the NHLBI’s centralized model suggests the possibility that a centralized data sharing model may increase IPD accessibility and use when compared with a decentralized model.

Notably, in our sample of 154 total trials across both institutes, only 16% of trials shared data, a rate that is quite low, although comparable with previous research^21–23^. It is important to highlight this low rate of data sharing from these large, resource-intensive trials, as they are publicly funded through the NIH and represent a significant public health investment. Through personal communication with officials at BioLINCC, we confirmed that there were difficulties with initial implementation of the data sharing policy, particularly some challenges tracking trials that were eligible for sharing, which may explain the low proportion of NHLBI-funded trials sharing data via BioLINCC. Personal communication with authors of other NHLBI-funded trials that did not share their data at BioLINCC confirmed that 1 study opted instead to share data via dbGap, an NIH depository for genomic data.

It is encouraging to note that NCI’s data sharing model has evolved since Febuary 2017 into NCTN/NCORP Data Archive – a centralized repository where data can be requested by any investigator, which is similar to BioLINCC’s data sharing model. It is expected that this shift to a centralized repository will increase the accessibility of these NCI-funded trials and benefit the broader research community. The NCTN/NCORP Data Archive will initially house NCTN trials that were published on or after January 1, 2015^18^.

Our findings demonstrate that the opportunity for generating more scientific knowledge through clinical trial data sharing is yet to be maximized and the TOPCAT trial, in which spironolactone failed to reduce the incidence of the composite primary outcome, is an illustrative example highlighting the importance and value of sharing data from large clinical trials. A large number of shared data publications have thus far resulted from the TOPCAT trial, many of which reanalyzed data regarding the primary outcome in the context of additional variables. This finding highlights the importance of sharing data, particularly when trial results are negative, as the potential exists for reinterpretation through additional analyses. It is noteworthy that the Final NIH Policy for Data Management and Sharing, released on October 2020, effective from January 25, 2023, indicated that the NIH expects that researchers will “maximize appropriate data sharing” when developing their Data Management and Data Sharing Plans, an important step towards more open science^13,14^.

The true value in sharing data lies in the generation and dissemination of new knowledge resulting from shared data, not only in making data available to be shared. This study characterized the “outcomes” associated with two data sharing models used by the NHLBI and NCI, finding low rates of data sharing and low numbers of shared data publications for the largest and most costly trials funded by the Institutes from 2010 to 2013. This study was a direct response to the Institute of Medicine’s call for attention to this issue^19^. However, while neither model resulted in the majority of trials being shared and both were associated with few shared data publications, the NHLBI’s centralized model was associated with greater data sharing, and a larger number of shared data publications, than the NCI’s decentrialized model. Future research is needed to further evaluate these and other existing data sharing models and initiatives to inform effective policies which maximize clinical trial data sharing, advancing the field’s understanding on how and to what extent IPD are being shared and used.

### Limitations

There are several limitations to our study. First, our study sample was limited to those trials expected to be of greatest importance to the clinical and research communities, as determined by trial costs and enrollment, limiting the total scope of this analysis. Further, we would expect rates of results reporting, publication, and data sharing, and use of those which shared data for secondary research, to be highest for these larger and more well-funded studies. As new NIH results reporting and data sharing policies are implemented, further research should examine if data are shared more readily and used more widely. Second, our assessments of publication and data sharing were conducted from a four-year period. It is possible that investigators later published or shared data from their trials. In fact, through personal communication with authors, investigators associated with one trial explained that the study was still ongoing and so had not yet been been published. Finally, the NCI data sharing model appeared to have been organized around Cooperative Groups, but not all NCI-funded trials included in our analysis were Cooperative Group trials. Nevertheless, all were still expected to be subject to the NIH data sharing policy.

## Methods

### Study sample – inclusion/exclusion

We assembled a sample of clinical trials sponsored by the NHLBI and NCI with a primary completion date between January 1, 2010 and December 31, 2013 and which had a project start date after May 1, 2006. We selected 2010–2013 as the primary completion date range to ensure that sufficient time had passed for investigators to publish their primary findings, deposit and share their data, and for external authors to request these data and undertake and publish their secondary analyses. May 1, 2006 was selected as the earliest project start date, as the NHLBI data sharing policy was extended to grant-supported studies in 2005.

Eligible studies were identified using data downloaded from ClinicalTrials.gov and NIH RePORTER. We selected studies that for which NIH listed total costs greater than or equal to $1,000,000 in any given fiscal year, ensuring we identified those which had direct costs greater than or equal to $500,000 in any given fiscal year and with more than 500 participants. After a preliminary search, there were only two studies that fit both criteria; we revised our search to also include studies which fit either the sample size or cost criteria.

Once eligible studies were identified, we matched studies sponsored by each Institute based on either cost and/or size.

### Main outcome measures

#### Sharing of individual patient-level data and results reporting

For NHLBI-sponsored trials, we determined whether IPD were available for request on the BioLINCC website in March 2021 and May 2022. From an initial sample of 14 NHLBI-funded trials, for those that were not listed on BioLINCC, we contacted NHLBI staff to determine if the data were available for sharing. We checked NCI’s new data sharing portal, NCTN/NCORP Data Archive, for data from the NCI-sponsored studies. For both NHLBI- and NCI-sponsored trials, we also checked ClinicalTrials.gov and the primary publication (if any) to find a link and/or any indication to shared data or intention to share data. We were conservative in our assessment of data sharing and thus did not request data or check the viability of links to shared data to confirm that data were truly shared and so we categorized all such trials as having shared data.

From an initial sample of 28 trials (14 trials per funding model), we contacted corresponding authors (or secondary and senior authors if we could not locate the email address of the corresponding author) of studies for which we could not identify shared data in those public data sharing platforms, to confirm whether they had shared their data with other investigators. An initial email was sent, with a follow-up email if we did not hear back from author(s), in March 2021. No further follow-up emails were sent and we did not look for alternative email addresses when emails bounced back.

We determined whether results were reported on ClinicalTrials.gov for all trials in March 2021 and April 2023.

### Publication identification

#### Primary publication

We defined primary publications as those reporting the results for the primary outcome (i.e. the outcome used to determine sample size and study design) in a peer-reviewed publication. Primary publications were identified from the linked NIH RePORTER data. For trials without a primary publication listed, between September 2020 and March 2021 and March-May 2022, we searched ClinicalTrials.gov, MEDLINE, Google Scholar and a Google search first using the trial’s NCT number, then the title of the clinical trial. From a sample of 28 trials (14 trials per funding model), those without an identifiable primary publication corresponding authors (or secondary and senior authors if we could not locate the email address of the corresponding author) were contacted to request a copy of the trial’s primary publication. An initial email was sent, with a follow-up email if author(s) did not respond to our request, in March 2021. No further follow-up emails were sent and we did not look for alternative email addresses when email(s) bounced back.

#### Secondary publications: “secondary internal publications” and “shared data publications”

We defined secondary publications as those reporting IPD analyses, in a peer-reviewed publication, that were separate from that reported in the primary publication. To identify secondary publications, during two separate periods of time: September 2020 and March 2021 and May 2022 to May 2023, we searched MEDLINE for all citations of the primary publication of the trials’ that shared data, manually screening the titles and abstracts to flag eligible studies. Full-text articles of flagged citations were reviewed for eligibility. We excluded publications reporting the results of pilot studies, feasibility studies, study protocols, interim analyses, and non-human studies. We defined shared data publications as those secondary publications reporting IPD analyses that were separate from that reported in the primary publication, authored only by external authors. Even if only one author from the primary publication was included on the secondary publication, we categorized the study as secondary internal publication, not a shared data publication.

### Impact

For each primary publication, we extracted the authorship, publishing journal, and intervention type (i.e., interventional or observational). For each secondary publication, we extracted the type of analysis (i.e. extended follow-up, reanalysis of primary outcome, secondary outcome reporting, subgroup analysis, pooled analysis, predictive analysis, meta-analysis), authorship, and publishing journal. For those trials with shared data publications, for both the secondary internal and shared data publications we extracted the citation counts, h-index of the publishing journal, abstract views, and the Altmetric Attention Score, an indicator of the amount of attention the articles have received.

A.R.F., A.D.Z. and S.S. identified eligible trials; A.R.F. and S.S. identified primary publications, A.R.F. identified shared data; A.R.F. and M.G. determined secondary internal and shared data publications; A.R.F., M.G. and A.O.E. extracted study type data; and A.R.F., S.S. and A.O.E. abstracted impact measure data. To ensure accuracy, an initial sample of data was verified externally prior to this study’s major revision. Throughout the data collection process, a random sample of abstracted data was periodically examined by a senior author (J.S.R.) to assess accuracy.

### Statistical methods

We calculated odds ratios to determine whether funder data sharing model was associated with data sharing and the generation of shared data publications. We used descriptive statistics to characterize the number of trials that published their findings, reported their results, shared their IPD, and had secondary publications resulting from shared data (external or internal). We also calculated the median and interquartile range (IQR) for the time from primary study completion to primary publication for those trials that published their findings. For those trials that had shared data publications, we characterized the type of studies conducted and calculated the median and IQR for number of secondary publications, citation counts, h-index of publishing journal, abstract views and Altmetric Attention Score. All analyses were conducted using Microsoft Excel, Version 16.59.

## Data Availability

The data generated from this study are publicly available at the Open Science Framework: 10.17605/OSF.IO/T8B6G^20^.

## Code availability

There is no custom computer code used to analyze the data generated from this study; all analyses were conducted using Microsoft Excel, Version 16.59.
